# First person – Manh Tin Ho and Jiongming Lu

**DOI:** 10.1242/dmm.048955

**Published:** 2021-03-18

## Abstract

First Person is a series of interviews with the first authors of a selection of papers published in Disease Models & Mechanisms, helping early-career researchers promote themselves alongside their papers. Manh Tin Ho and Jiongming Lu are co-first authors on ‘A translation-independent activity of PheRS activates growth and proliferation in *Drosophila*’, published in DMM. Manh Tin conducted the research described in this article while a PhD student in the lab of Beat Suter at the University of Bern, Bern, Switzerland, and is now a postdoctoral researcher in the lab of Daniel Fuster at the University of Bern, investigating kidney disease and sodium/proton exchange membrane proteins. Jiongming conducted the research described in this article while a PhD student in the lab of Beat Suter at the University of Bern, and is now a postdoctoral researcher in the lab of Linda Partridge at the Max Planck Institute for Biology of Ageing, Köln, Germany, investigating molecular mechanisms and new strategies of healthy ageing.


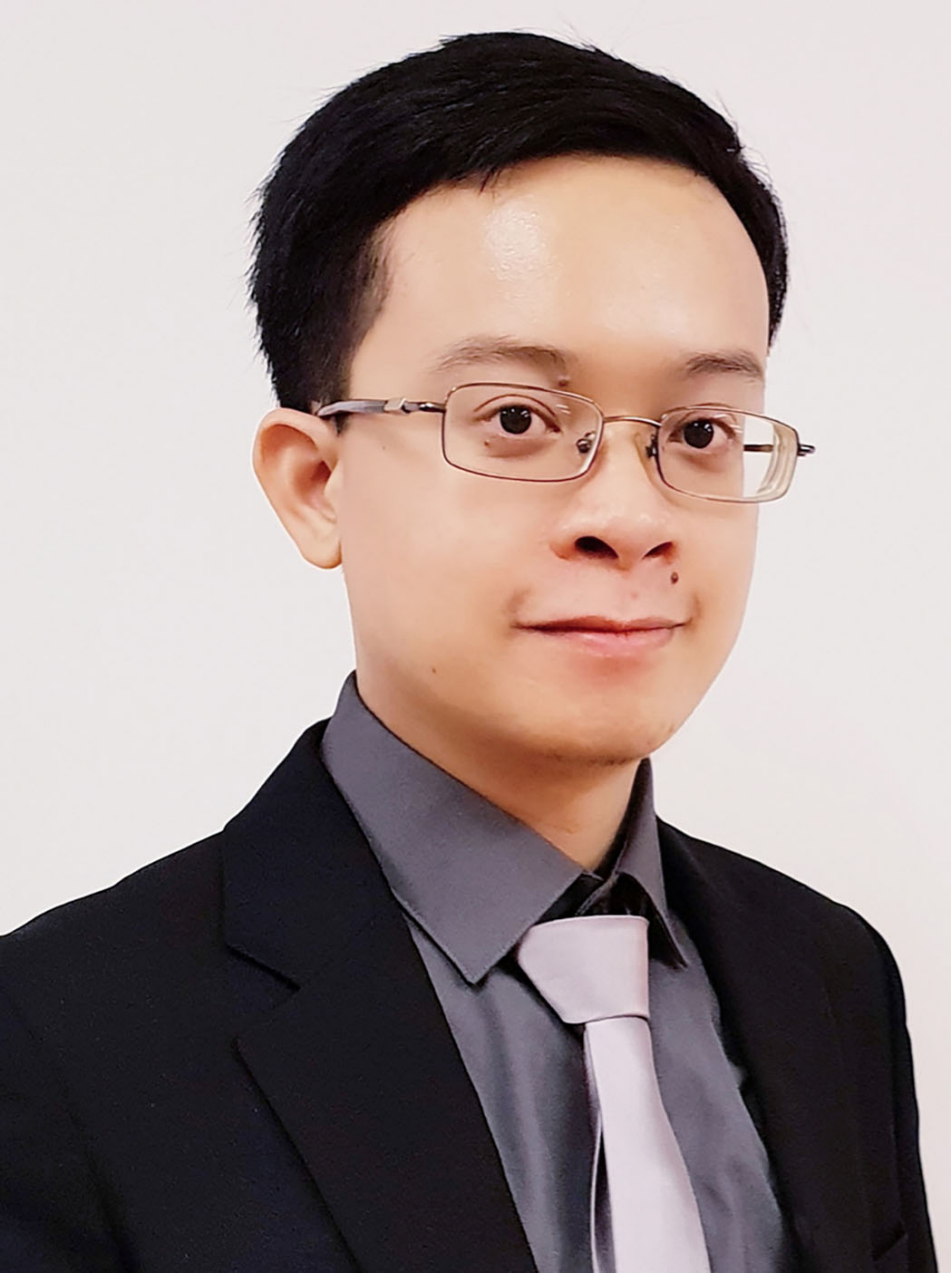


**Manh Tin Ho**


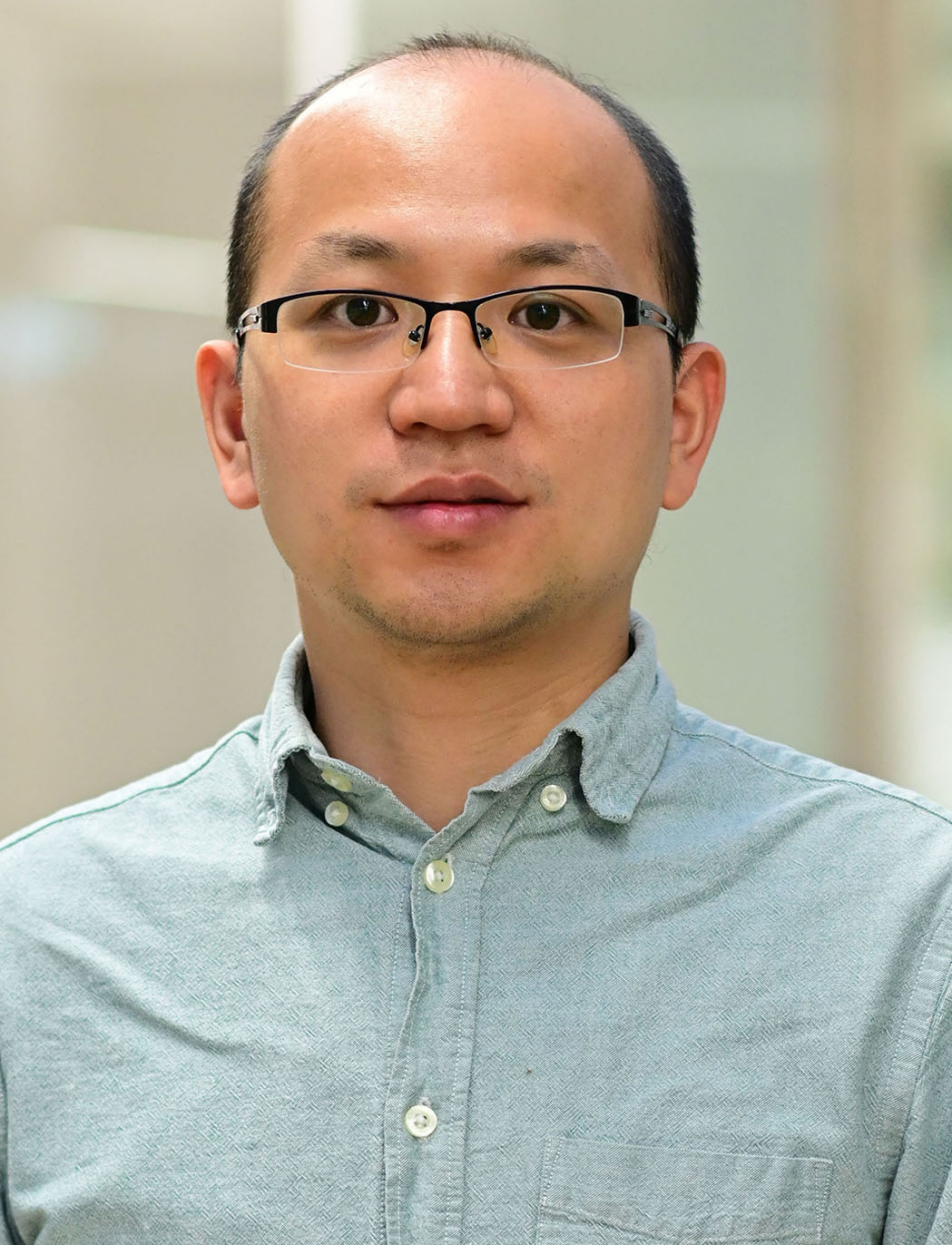


**Jiongming Lu**

**How would you explain the main findings of your paper to non-scientific family and friends?**

**MTH:** Aminoacyl transfer RNA (tRNA) synthetases (aaRSs) are well known for their simple roles in the ligation of amino acids to their cognate tRNAs. Recently, more functions of these enzymes in regulating additional biological processes have been discovered. We have found that Phenylalanyl tRNA synthetase (PheRS), one of the most complex aaRSs, can induce increased cell proliferation when its α-subunit is overexpressed. This matches well with the fact that PheRS (FARS) was found at a higher level in most cancer tissues. Surprisingly, this proliferative effect of PheRS is independent of the main function in aminoacylation. We made mutant PheRS, which is unable to ligate phenylalanine to the tRNA; however, our mutant version still caused the proliferative effect. We, therefore, conclude that PheRS can play this additional function in cells and could be a potential target for cancer studies.

**JL:** aaRSs are a family of ubiquitously expressed enzymes and their aminoacylation function is essential for protein synthesis and thus also for the survival of all cells. Recent evidence has shown that PheRS is highly expressed in cancers. It sounds reasonable as cancer cells synthesize more proteins and hence need a larger amount of PheRS. However, by using the animal model fruit fly *Drosophila*, we show that PheRS can also be a potential causal factor for cancers. Our study reveals that high levels of PheRS can increase cell proliferation, and, moreover, the α-subunit of PheRS, which is clearly unable to perform the aminoacylation function on its own, is sufficient for the growth effect. Our finding illustrates that the ancient enzyme has a non-canonical function, and also provides a novel target for cancer treatment.

**What are the potential implications of these results for your field of research?**

**MTH:** Among the aaRS enzymes, PheRS has a very complex structure that combines two pairs of each subunit (α and β). This makes it one of the most difficult aaRSs to study because one has to solve the relationship between the two subunits for every phenotype explored. By applying the fruit fly model *Drosophila*, we can independently investigate each subunit in separate tissues or in the whole body. With its powerful genetic tools, we can also take advantage of well-developed tools such as expressing a gene in only half of the wing disc or in clonal twin spots to study cell proliferation with an internal control at the same tissues. Taken together, we found that the α-subunit is crucial for cell proliferation and this subunit can be involved in other cellular processes independent of the β-subunit.

**JL:** First, our research shows that *Drosophila* is a great model system to study the non-canonical function of these highly conserved proteins. The advantages of this system will be further described later in this interview. Furthermore, our results demonstrate that elevated levels of PheRS can drive cell proliferation and growth, indicating that PheRS can be a potential target for the treatment of certain types of cancers.

**What are the main advantages and drawbacks of the model system you have used as it relates to the disease you are investigating?**

**JL:** We use *Drosophila* as our model organism and this system exhibits many advantages. The powerful genetic tools make it possible to express genes of our interest in almost any tissues and at any time. These tools are available in different stock centres or can be easily requested from researchers in the field. Although flies are tiny, they have various tissues that are ideal for studying cell proliferation and growth; for example, wing discs, follicle cells, eye cells and gut stem cells. The main drawback is that their tissues are more homogenous in the composition of cell types compared with mice or human cancers, so considerations need to be taken when translating these findings to humans.

**What has surprised you the most while conducting your research?**

**MTH:** We were extremely surprised when we found that the α-PheRS mutant is stably expressed and still induces cell proliferation. Even though this mutant is not able to perform the aminoacylation function, it can still bind to the β-subunit of PheRS. Furthermore, this subunit can survive alone and exhibit proliferative effects. This is highly unexpected, because recent studies have shown that both subunits have to exist and co-stabilize each other. This is also exciting as it confirms that this function is not related to aminoacylation, so it is indeed a non-canonical function. Additionally, it was not in line with our expectation, as we previously hypothesized that this non-canonical function may be contributed by a non-essential domain in the β-subunit of PheRS. These kinds of unexpected surprises are some of the most rewarding aspects of science.

“[…] elevated levels of PheRS can drive cell proliferation and growth, indicating that PheRS can be a potential target for the treatment of certain types of cancers.”

**Describe what you think is the most significant challenge impacting your research at this time and how will this be addressed over the next 10 years?**

**MTH:** Most researchers that I have met are surprised that people still perform research on ‘old, classical’ models like *Drosophila*. Many also question whether there is something new in this model to do or whether this model can solve the complicated diseases in humans. The focus is now more on studies on higher model animals, like mice. This makes fewer people go into this field and means that less funds are allocated, leading to long-term problems for studies by the fly community.

**JL:** In my opinion, the most significant challenge at this time is that less and less funding goes to simple model organisms. Simple model organisms – including yeast, worm, flies and fish – have made extreme contributions to various basic knowledge and concepts in modern life sciences. However, now they have a difficult time securing enough funding, as funding agencies prefer so-called higher organisms. A model organism is just a ‘model’; it should be judged by whether it suits a specific scientific question or not, not by whether the organism is a lower or higher one.

**Figure:**
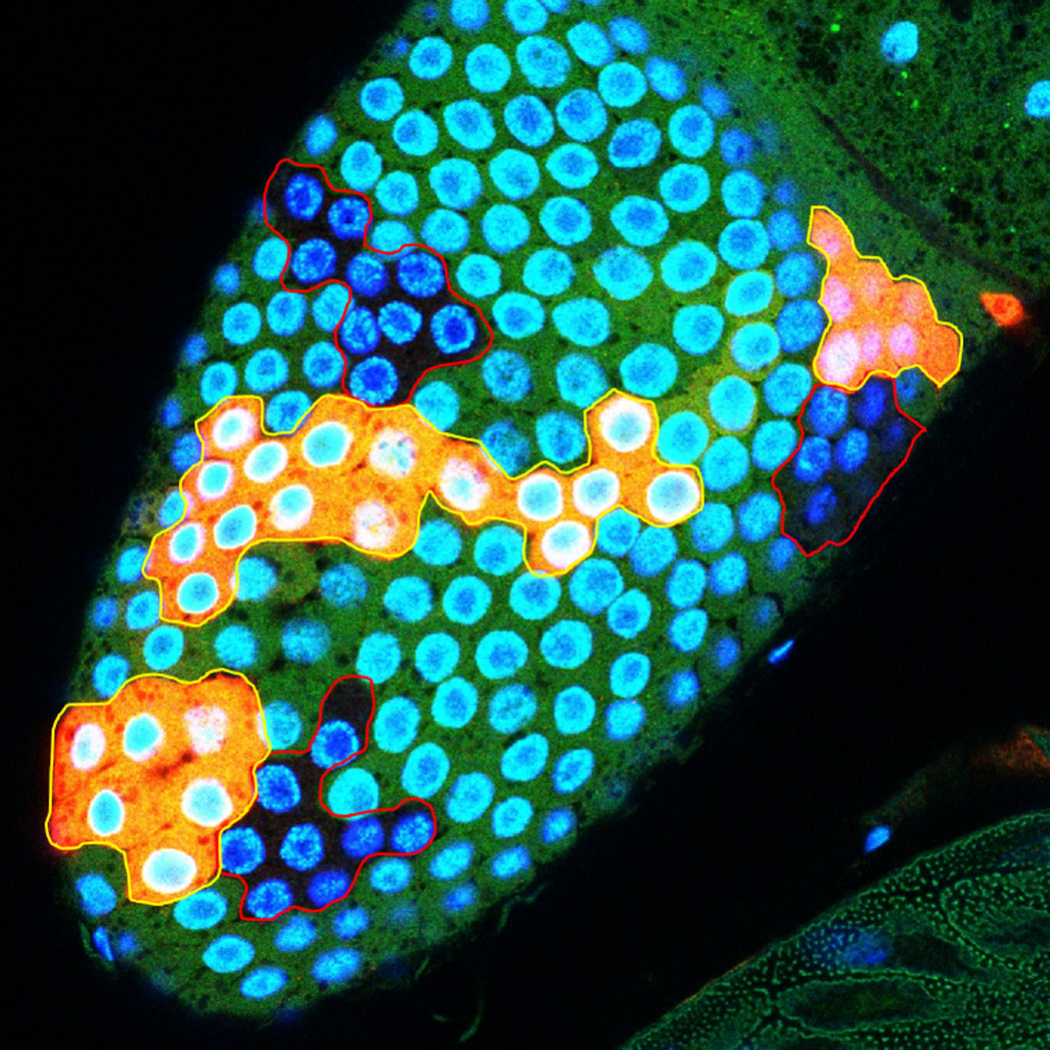
**Twin-spot clones in follicle cells of the fly ovary (blue clones with red outline, wild-type clones – internal control; orange clones with yellow outline, overexpressing clones).**

**What changes do you think could improve the professional lives of early-career scientists?**

**MTH:** In my opinion, the distribution of funding with a higher preference to young early-career scientists is very important. No matter whether at the stage of a postdoctoral researcher or as an independent investigator, we always experience discrimination in race, gender, language. These issues should be avoided in the science community. Presently, they are still obstacles that prevent young scientists from approaching their professional careers.

**JL:** I think the change that could contribute best is to increase funding opportunities to early-career scientists, including for the postdoc stage and the transition stage to an independent group leader. Many breakthrough findings are from small and early-career groups, not large collaboration projects. For early-career scientists, it is also very beneficial to attend training in project management, communications, leadership and teaching skills. A good mentor also helps a lot in career development.

“A good mentor helps a lot in career development.”

**What's next for you?**

**MTH:** I recently started my postdoctoral training and I am very interested in applying what I have studied in my PhD time to solve the questions in human diseases. I joined a group at the Insel hospital, the biggest hospital in Bern, where I feel very near to patients and also have more motivation to work hard and help improve human health. I am also particularly interested in microscopy and love to contribute the knowledge I have learned at the Microscopy Imaging Center at the University of Bern to explore super-resolution imaging.

**JL:** I am now in a transition stage that could move me forward to an independent investigator position. The most important ‘next’ for me is to find a faculty position, which would allow me to use the animal model fruit flies to study nutrients and ageing. In the long-term, I also would like to pursue new model organisms that can address specific scientific questions for humans.
